# Suppression of isoprenylcysteine carboxylmethyltransferase compromises DNA damage repair

**DOI:** 10.26508/lsa.202101144

**Published:** 2021-10-05

**Authors:** Jingyi Tang, Patrick J Casey, Mei Wang

**Affiliations:** 1 Duke-NUS Medical School, Program in Cancer and Stem Cell, Singapore, Singapore; 2 Department of Pharmacology and Cancer Biology, Duke University School of Medicine, Durham, NC, USA; 3 Department of Biochemistry, National University of Singapore, Singapore 117596

## Abstract

DNA damage is a double-edged sword for cancer cells. On the one hand, DNA damage–induced genomic instability contributes to cancer development; on the other hand, accumulating damage compromises proliferation and survival of cancer cells. Understanding the key regulators of DNA damage repair machinery would benefit the development of cancer therapies that induce DNA damage and apoptosis. In this study, we found that isoprenylcysteine carboxylmethyltransferase (ICMT), a posttranslational modification enzyme, plays an important role in DNA damage repair. We found that ICMT suppression consistently reduces the activity of MAPK signaling, which compromises the expression of key proteins in the DNA damage repair machinery. The ensuing accumulation of DNA damage leads to cell cycle arrest and apoptosis in multiple breast cancer cells. Interestingly, these observations are more pronounced in cells grown under anchorage-independent conditions or grown in vivo. Consistent with the negative impact on DNA repair, ICMT inhibition transforms the cancer cells into a “BRCA-like” state, hence sensitizing cancer cells to the treatment of PARP inhibitor and other DNA damage–inducing agents.

## Abstract (precis)

Inhibition of isoprenylcysteine carboxylmethyltransferase reduces cancer cells’ ability to repair DNA damage by suppressing the expression of critical DNA damage repair pathway genes, hence increasing their vulnerability to DNA damaging insults such as PARP inhibitors and other DNA damage agents.

## Introduction

DNA damage response plays important roles in cancer development and is a major focus of attention in cancer therapy (1, 2). DNA damage, which can be the result of radiation (3, 4), drugs (5), oxidative stress (6), or replicative stress (7, 8), triggers DNA repair pathways and cell cycle check points and can ultimately lead to programmed cell death (9, 10, 11). The resilience and adaptation to DNA damage–induced genomic instability contributes to cancer development (12); many cancers arise because of an impairment of the DNA damage repair machinery and associated genomic instability (13, 14). Interestingly, this impairment of DNA damage repair becomes a vulnerability when overwhelmed, forming the rationale for targeted therapy using DNA damage–inducing agents for selected cancers (15). A prime example is inhibition of poly ADP-ribose polymerase 1 (PARP1), an enzyme important for the DNA repair in many ways, with particular involvement in repairing single-strand breaks (16, 17). Inhibition of PARP activity can lead to the accumulation of DNA double-strand breaks in proliferating cells (18). The cancers that have existing deficiencies in DNA repair machinery, such as those with BRCA1/2 mutations (19, 20) or PTEN dysfunction (21), are particularly vulnerable to PARP inhibition because they rely more heavily on the single-strand break repair pathway in which PARP1 is a critical component. In contrast, cancer cells that have intact or elevated DNA repair capacity are significantly more resistant to PARP1-targeting agents (22, 23, 24, 25). For these cancers, PARP inhibitors have been used in combination with other targeted therapy to increase efficacy (26, 27, 28, 29, 30). The ever-expanding efforts to understand the multifaceted regulation of DNA damage repair are identifying novel and effective synthetic lethality combinations to increase the responsiveness of cancers, particularly those having efficient DNA repair machinery that are otherwise resistant to DNA damage–inducing approaches such as irradiation or PARP inhibitor (31, 32).

In this study we have found that isoprenylcysteine carboxylmethyltransferase (ICMT), an enzyme of the protein prenylation pathway, plays an important role in DNA damage repair. Protein prenylation is a posttranslational modification process that occurs in all eukaryotes (33). The three-step enzymatic process starts with the addition of an isoprenoid lipid to the cysteine residue at the C-terminal consensus sequences, usually composed of CAAX (cysteine, aliphatic, and any amino acid), by one of the protein prenyltransferases (34, 35). The C-terminal–AAX amino acids are then removed by the RCE1 endopeptidase (36, 37, 38). The last step is the cysteine carboxylmethylation catalyzed by ICMT, which completes the post-prenylation processing that regulates the functions of various substrate proteins, among them the oncogenic RAS GTPases (39, 40, 41, 42). In the past decade, studies have demonstrated that suppression of ICMT reduces tumorigenesis and cancer progression of various human cancers (42, 43, 44, 45, 46, 47). Although the complete mechanism of ICMT regulation of cell signaling is still an active area of investigation, it is clear that ICMT inhibition, either genetically or pharmacologically, affects the downstream signaling of ICMT substrates to regulate essential cell functions such as proliferation and survival (48, 49, 50).

The MAPK signaling cascade, also known as the Ras-Raf-MEK-ERK pathway, plays an important role in transducing extracellular signals to cellular response (51). Although it is often considered the most canonical downstream pathway of oncogenic RAS, MAPK signaling responds to many stimuli and has extensive cross-talk with other signaling components. As such, the MAPK pathway is affected by growth factors/cytokines, drugs, and irradiation, among others, to influence many cellular events, such as proliferation and survival (51). Although the involvement of MAPK signaling in DNA damage and DNA damage repair pathways have been reported for many cancers, its multifaceted roles needs to be further clarified, especially the manners in which the pathway contributes in different cellular contexts (52, 53, 54). In some situations, inhibiting ERK activation attenuates the DNA damage–induced cell death, suggesting a collaborative role of ERK in DNA damage or impairing DNA repair (55, 56, 57). On the other hand, it is reported that ERK signaling is very important for homologous recombination (HR) repair in response to DNA damage (58, 59); inhibiting ERK function was reported to sensitize cancer cells to radiation-induced DNA damage, cell cycle arrest, and cell death (60).

The current study demonstrates that ICMT is important for the DNA damage repair function through its regulation of MAPK signaling and its impact on the expression of key DNA repair proteins. Inhibition of ICMT reduces ERK activation, compromises DNA damage repair, leads to the accumulation of DNA damage both at baseline growth condition and in response to DNA damage–inducing agents, inhibits proliferation and ultimately induces apoptosis. Furthermore, ICMT inhibition substantially increases the sensitivity of breast cancer cells to PARP1 inhibitor treatment, offering a novel therapeutic approach in the treatment of this group of cancers.

## Results

### Loss of ICMT induces apoptosis and abolishes the ability of MDA-MB231 breast cancer cells to form colonies in soft agar and form tumors in vivo

**Figure 1.**
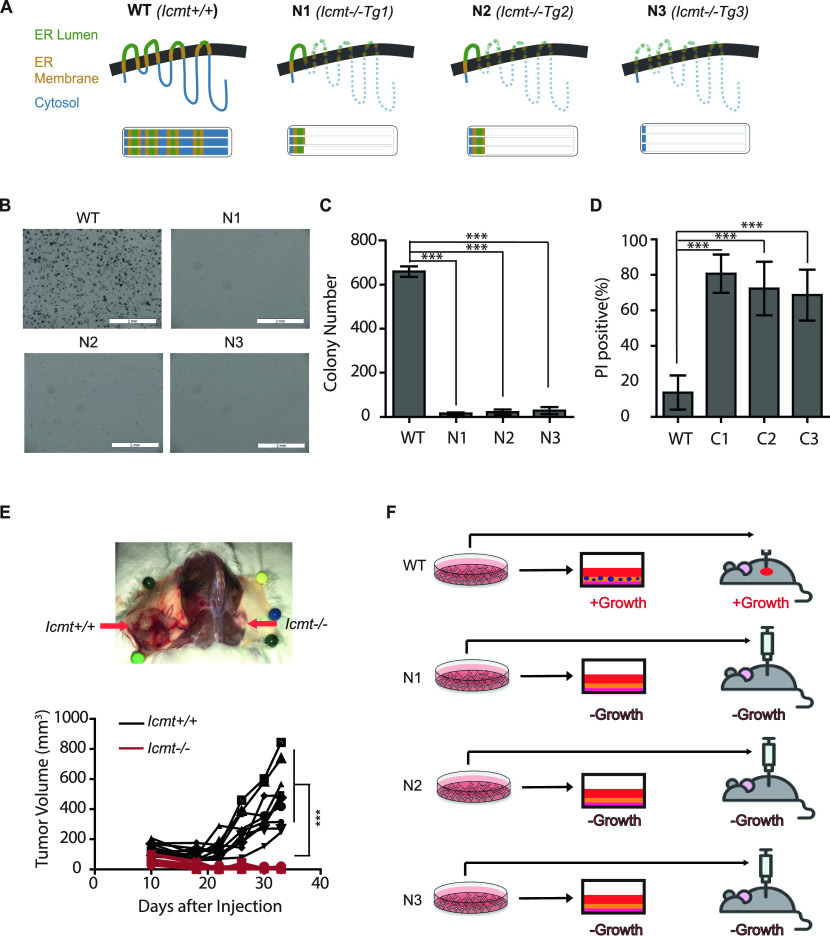
Loss of ICMT abolishes the ability of anchorage-independent growth in vitro and tumor formation of MDA-MB231 breast cancer cells. **(A)** Schematic illustration of the predicted ICMT polypeptide translated from the genomic DNA sequences in *Icmt*^*+/+*^ (*WT*) and predicted residual peptides in the three mixed clones of *Icmt*^*−/−*^ (*null*) MDA-MB231 cells, namely, N1, N2, and N3, generated from three different targeting sequences for the CRISPR-Cas9 editing, respectively. MDA-MB231 cells have three copies of the *Icmt* gene as illustrated in the lower part of panel (A). The blue-, gold-, and green-colored bars represent the cytosolic, endoplasmic reticulum transmembrane, and endoplasmic reticulum luminal regions of ICMT, respectively. The thick and thin dashed lines represent frameshifted nonsense polypeptides and the missing part of ICMT, respectively. The isogenic cell lines were generated by CRISPR/Cas9 gene editing. Each of these cell lines (WT, N1, N2, and N3) is a mixture at the same ratio of three individual clones generated using the same targeting sgRNA (Tg1, Tg2, and Tg3, respectively). **(B)** Soft agar colony formation assay for *Icmt*^*+/+*^ (WT) and *Icmt*^*−/−*^ (N1, N2 and N3) isogenic cell lines. For each cell line, 100,000 cells were seeded in 0.25% noble agar and cultured for 2–3 wk before staining by MTS at 0.2 mg/ml. The colonies were then imaged by Olympus SZX16 Research Stereo Microscope; scale bar length = 2 mm. **(C)** Colonies in (B) were quantified by openCFU and presented using Excel; at least 10 images were analyzed for each condition. “***”*P* < 0.0005. The experiment was performed three times with similar results. **(D)** Apoptotic cells were quantified using an in-soft-agar propidium iodide (PI) staining method. The cells of WT, N1, N2, and N3 were seeded in soft agar the same way as in colony formation assay. 3 d after the seeding, the media above the agar was replaced by 10 μg/ml PI in PBS and incubated for 30 min, followed by fluorescent imaging for PI using Olympus IX71S1F3 fluorescent Microscope. Total and PI-positive cells were quantified by OpenCFU and analyzed by Prism5; the percentage of PI-positive cells for each condition is presented. “***”*P* < 0.0005. The experiment was performed three times with similar results. **(E)** Tumor formation ability of *Icmt*^*+/+*^ and *Icmt*^*−/−*^ isogenic cells in a xenograft model. Top: representative mouse image to show the difference in tumor growth of cells with and without the presence of ICMT; bottom: tumor growth graph of *Icmt*^*+/+*^ and *Icmt*^*−/−*^ cells from eight mice. **(F)** Schematic of the growth phenotypes of *Icmt*^*+/+*^ and *Icmt*^*−/−*^ cells in adherent culture, soft agar colony formation condition and in the in vivo model. Individual black line or red line represents a tumor derived from *Icmt*^*+/+*^ cells or *Icmt*^*−/−*^ cells, respectively.

**Figure S1.**
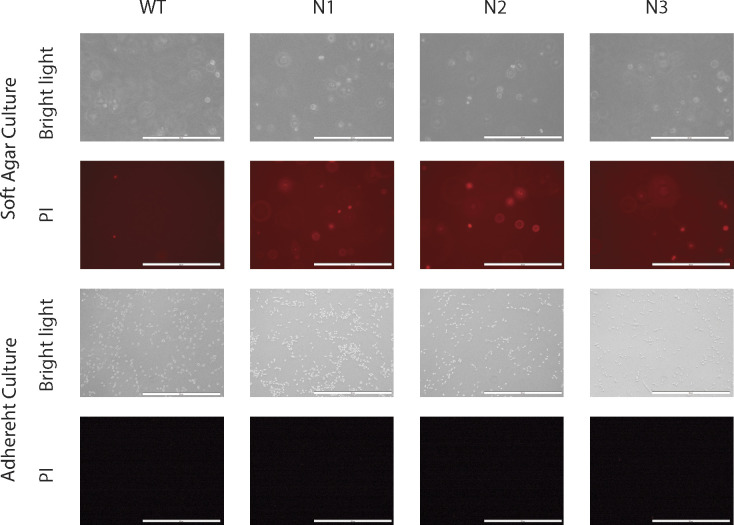
Propidium iodide (PI) staining to identify apoptotic cells in soft agar and in adherent culture conditions. Same numbers of *Icmt*^*+/+*^ and *Icmt*^*−/−*^ (N1, N2, and N3) MDA-MB231 isogenic cells were seeded either in soft agar or normal adherent culture conditions. 3 d after the seeding, the medium above the agar was replaced by 10 μg/ml PI in PBS and incubated for 30 min, followed by light and fluorescent imaging for PI using Olympus IX71S1F3 fluorescent Microscope. The scale bar length is 500 μm. Top two rows: cells in soft agar; bottom two rows: cells in adherent culture. The experiment was performed three times with similar results.

To study the impact of ICMT suppression on tumorigenesis, we have generated, from parental MDA-MB231 cells, *Icmt* wild type, and multiple loss-of-*Icmt* isogenic cell lines, which we designated *Icmt*^*+/+*^ (WT) clones, and N1, N2, and N3 *Icmt*^*−/−*^ mixed clones (generated by guide RNA sequence 1, 2, and 3, respectively) (Fig 1A). MDA-MB231 cells have three copies of the ICMT gene, and the genotypes and the remnants of ICMT polypeptide of the coding sequences following CRISPR-mediated deletion are shown in Fig 1A. It is apparent from genomic DNA sequencing that the *Icmt*^*−/−*^ clones produce no functional ICMT protein because the C-terminal region of the protein that contains the catalytic domain is completely missing in the null cells (44). In the soft agar colony formation study typically used to assess transformation, we found that, whereas the *Icmt*^*+/+*^ cells form colonies readily, all the *Icmt*^*−/−*^ clones lost their ability to grow (Fig 1B and C), suggesting that ICMT is essential for the ability of MDA-MB231 cells to proliferate under an anchorage-independent condition—the defining feature of malignant cells. In-agar propidium iodide (PI) staining showed that the *Icmt*^*−/−*^ cells placed in soft agar underwent massive apoptosis (Figs 1D and S1), whereas under the adherent condition there was no significant elevation in apoptosis for *Icmt*^*−/−*^ cells compared to the *Icmt*^*+/+*^ cells (Fig S1). The in vivo tumor formation abilities of the *Icmt*^*+/+*^ and *Icmt*^*−/−*^ MDA-MB231 cells are consistent to their ability to form soft agar colonies (Fig 1E), which is the expected result as the anchorage-independent growth is the in vitro study of choice in the assessment of malignant transformation. The model in Fig 1F summarizes the essential role of ICMT in cancer cell colony formation and tumor formation, two properties that distinguish benign and malignant cells.

### ICMT inhibition causes G2/M cell cycle arrest

**Figure 2.**
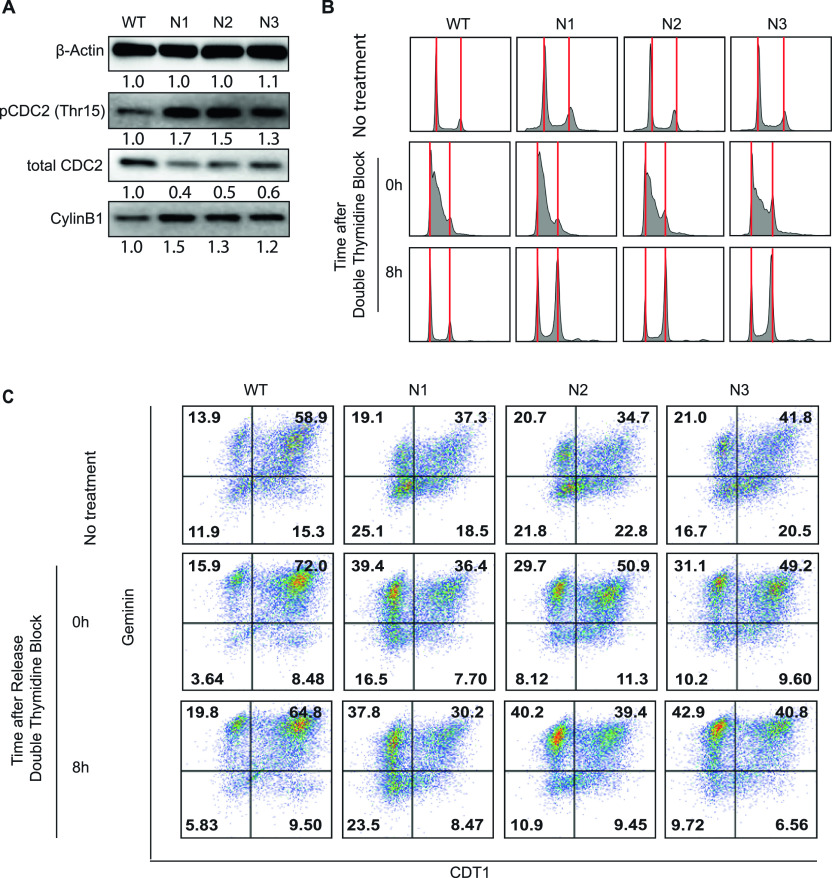
Loss of ICMT leads to G2-M cell cycle arrest. **(A)** Immunoblot analysis for the G2-M cell cycle markers: pCDC2(T15), total-CDC2, and cyclin B1 on the *Icmt*^*+/+*^ (WT) and *Icmt*^*−/−*^ (N1, N2, and N3) isogenic cell lines harvested after growing under standard culture conditions before reach confluency and processed for SDS–PAGE. The numbers below each marker are the densitometry quantification of band intensity. **(B)** Cell cycle distribution of the isogenic cells in the baseline state without synchronization (top row), and following double thymidine synchronization (0 h, middle row) and 8 h post-release (bottom row). The red vertical lines mark the G1 and G2/M peaks. The cells were prepared by standard propidium iodide staining method for cell cycle analysis, as described in the Materials and Methods section. **(C)** Flow cytometry analysis of Citrine-Geminin and mCherry-Cdt1 in the isogenic cell lines stably expressing these cell cycle markers. Analysis was performed at the baseline state without synchronization (top row), at the time of release from the double thymidine block (middle row, 0 h), and 8 h after release from double thymidine block (bottom row). **(B)** These cells were subjected to the same preparation conditions as in (B) before standard flow cytometry analysis. The experiments were performed three times with similar findings.

In addition to increased apoptosis, we found that loss of ICMT function resulted in the increase of pCDC2(Thr15) and cyclin B1 levels, consistent with cell cycle arrest at the G2/M phase (Fig 2A) (61, 62). To investigate this phenomenon further, we performed DNA content flow cytometry analysis on the *Icmt*^*+/+*^ and *Icmt*^−/−^ cells both at baseline and at 0 and 8 h following release from double thymidine block. Double thymidine block stops cell cycle progression at the G1/S phase; subsequence release allows observation of synchronized cell cycle progression. Flow cytometry analysis showed that the *Icmt*^−/−^ N1, N2 and N3 cells have significant G2/M arrest, which is especially apparent for the cells 8 h after the release from double thymidine block, at which point *Icmt*^*+/+*^ cells are almost back to the normal cell cycle distribution (Fig 2B).

To observe the dynamic cell cycle changes in liver cell populations, we established stable cell lines from *Icmt*^*+/+*^ and *Icmt*^*−/−*^ cells that expressed fluorescent cell cycle indicator proteins Citrine-Geminin and mCherry-Cdt1. Geminin and Cdt1 are two proteins whose degradation are regulated in cell cycle dependent manner (63). Therefore, the relative intensities of the fluorescent protein-tagged Geminin and Cdt1 allow identification of cell populations in different phase of cell cycle. At the G1 to S transition, both proteins are present, so the right upper quadrant in the flow diagram indicates the G1/S cells. The G1 cells and G2/M cells express Cdt1 and Geminin, respectively; hence they are represented in the right and upper left quadrants. The baseline distribution analysis showed that, in comparison to *Icmt*^*+/+*^ cells, *Icmt*^*−/−*^ cells accumulate at G2/M phase (Fig 2C). To carefully examine the differences in cell cycle distribution between the *Icmt*^*+/+*^ and *Icmt*^*−/−*^ cells using these fluorescent reporters, we again preformed double thymidine synchronization and release analysis. At the time of release from synchronization, the *Icmt*^*+/+*^ cells accumulated at the G1/S transition point (Cdt1+/Geminin+ and Cdt1−/Geminin+), as expected. In contrast, the double thymidine treatment was less effective in synchronizing the *Icmt*^*−/−*^ cells at the G1/S phase—a significant number of cells were observed at the G2/M stage (Cdt1−/Geminin+) (Fig 2C). At 8 h after releasing from the block, we observed that *Icmt*^*−/−*^ cells continue to be arrested at G2/M phases, whereas the *Icmt*^*+/+*^ cells had essentially re-established the baseline distribution (Fig 2C). This multimodality analysis supports the conclusion that loss of ICMT function leads to aberrant cell cycle progression, particularly notable for G2/M arrest.

### ICMT loss of function leads to accumulation of DNA damage and apoptosis, which are the results of impaired DNA damage repair

**Figure 3.**
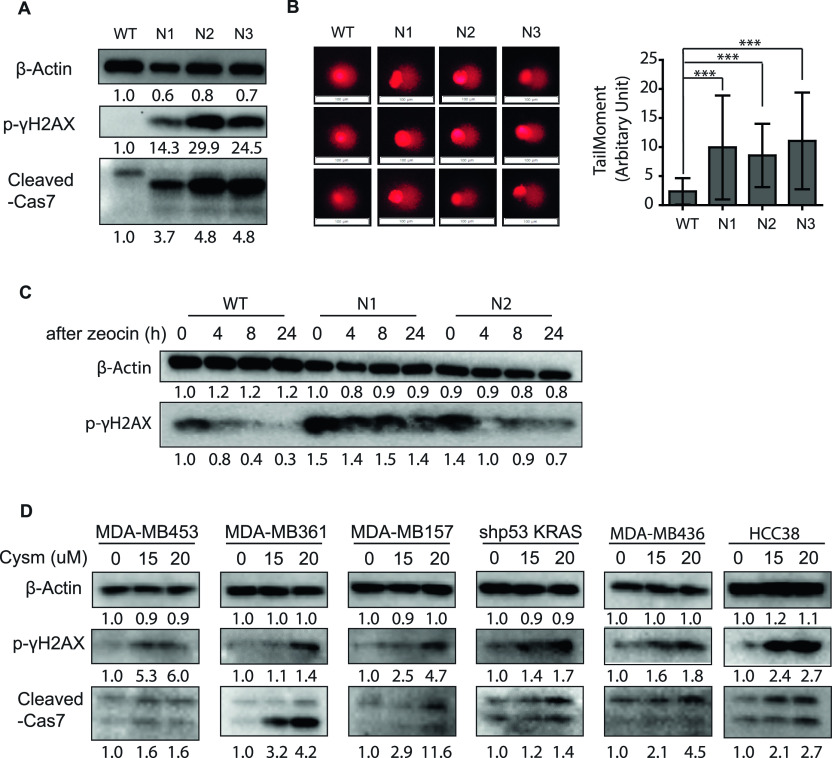
Loss of ICMT results in the accumulation of DNA damage. **(A)** Immunoblot analysis of DNA damage and apoptosis marker proteins in *Icmt*^*+/+*^ (WT) and *Icmt*^*−/−*^ (N1, N2 and N3) isogenic cell lines. Lysates were prepared from cells seeded in soft agar culture and grown for 3 d; the preparation method is described in detail recently (104). **(B)** Comet assay for DNA damage in the isogenic cell lines. Cells were cultured in low attachment plates for 3 d before prepared for the comet assay detailed in the Materials and Methods section (67). DNA in the cells was visualized by propidium iodide staining and imaged by Olympus IX71S1F3 fluorescent microscope; three representative cells are shown for each isogenic cell line (top panel). Scale bar length = 100 μm. The right side of the panel shows the result of analysis of more than 100 cells for each cell line using the Casplab (https://casplab.com/download) software to calculate the TailMoments, which quantify the extent of DNA damage. “***”*P* < 0.001. The study was repeated with similar results. **(C)** Immunoblot analysis of lysates from the isogenic cell lines for the DNA damage marker p-γH_2_AX. Cells grown in normal culture were treated with 1 μg/ml zeocin for 48 h, followed by recovery in fresh drug-free medium for 0, 4, 8, and 24 h before lysed and prepared for SDS–PAGE and immunoblot analysis for the indicated proteins. **(D)** Immunoblot analysis on several breast cancer cell lines to study the impact of ICMT inhibitor treatment on DNA damage and apoptosis. For all immunoblots, the numbers below each marker are the densitometry quantification of band intensity.

One of the major causes of prolonged G2/M arrest and apoptosis in cells is DNA damage, hence we evaluated markers of DNA damage in the *Icmt*^*+/+*^ and *Icmt*^*−/−*^ cells. Immunoblot analysis on the cells collected from soft agar growth condition showed that, in comparison to the *Icmt*^*+/+*^ cells, the *Icmt*^*−/−*^ cells had elevated p-γH2AX, accompanied by increased cleaved caspase 7 (Fig 3A), which suggested increased DNA damage and the programmed cell death (64). The comet assay is commonly used to measure DNA damage at the single cell level by visualizing the migration of DNA fragments in gel electrophoresis by fluorescence microscopy. The “tail” that trails the nucleus in the fluorescent images can be quantified using Casplab software (65) (https://casplab.com/download), and the extent of DNA damage is expressed as the “TailMoment” (66, 67). In the comparative comet assay study, we found that *Icmt*^*−/−*^ cells have significantly bigger TailMoment than the *Icmt*^*+/+*^ cells (Fig 3B). We postulated that the apparent increase in DNA damage observed in the *Icmt*^*−/−*^ cells was either the result of increased DNA damage or of diminished capacity for DNA damage repair. To distinguish between these two causes, we first treated both the *Icmt*^*+/+*^ and *Icmt*^*−/−*^ cells with zeocin (phleomycin D1), an irradiation mimic agent, to generate DNA damage, followed by a recovery phase in drug-free medium to allow cells to repair the DNA damage. Immunoblot analysis for p-γH2AX level was used to follow the repair during the recovering phase. We found that, even though there was no consistent difference in p-γH2AX levels between the *Icmt*^*+/+*^ *and Icmt*^*−/−*^ cells at the time of zeocin removal, the level of p-γH2AX persisted in the *Icmt*^*−/−*^ cells but was reduced rapidly in the *Icmt*^*+/+*^ cells during recovery, which suggests that loss of ICMT function compromises the cells’ ability for DNA damage repair (Fig 3C) (64). To evaluate whether the role of ICMT in regulating DNA damage repair is limited only to MDA-MB231 cells, we treated several breast cancer cell line with ICMT small molecule inhibitor cysmethynil (49, 68). We found that ICMT inhibitor treatment led to elevated levels of the DNA damage marker p-γH_2_AX and cleavage of caspase 7 in a range of breast epithelial cancer cells (Fig 3D), consistent with the observation on the MDA-MB231 *Icmt*^*−/−*^ cells. These data illustrate that ICMT is involved in the regulation of DNA damage repair in breast epithelial cancer cells with varied genetic background.

In conclusion, multimodality analyses support the notion that loss of ICMT results in defective DNA repair, and that the lingering DNA damage delays cell cycle entry at G2/M check points and leads to apoptotic cell death.

### DNA damage repair requires robust MAPK signaling

**Figure 4.**
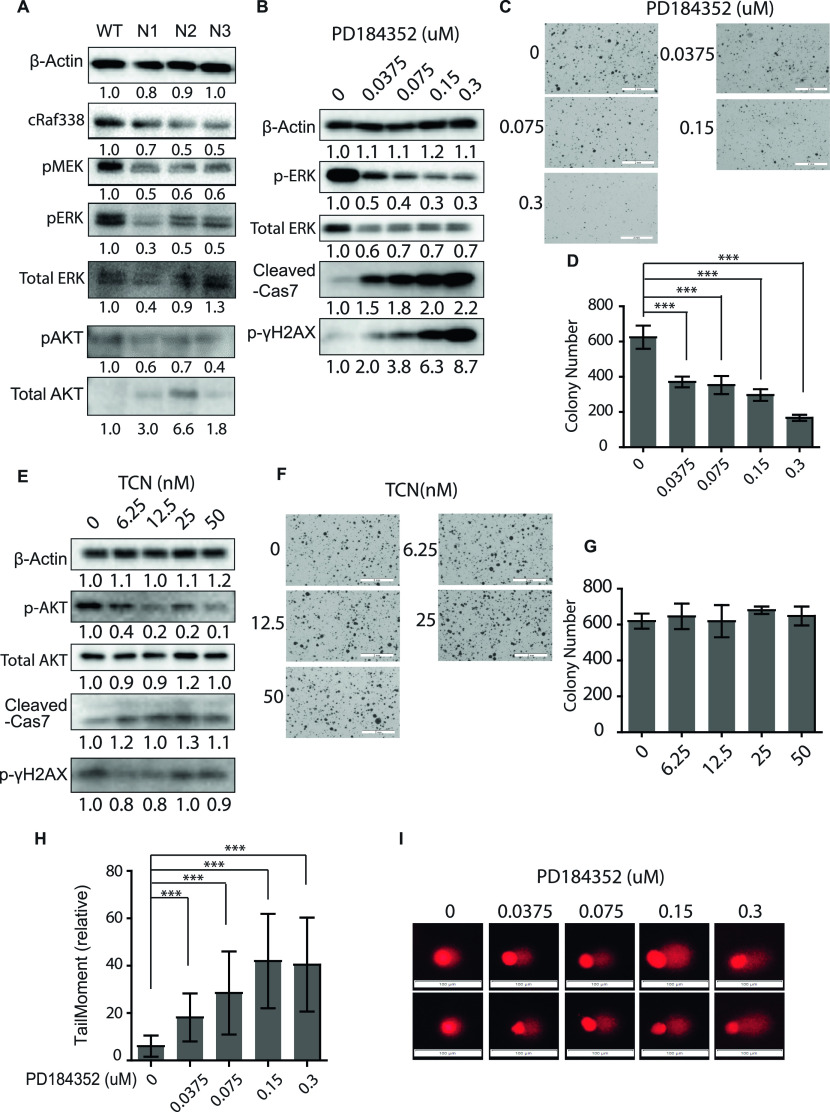
Maintenance of a robust MAPK signaling is necessary for DNA damage repair in MDA-MB231 breast cancer cells. **(A)** Immunoblot for the MAPK pathway proteins—pRAF, pMEK, and pERK—and PI3K downstream proteins pAKT and AKT in the lysates of *Icmt*^*+/+*^ (WT) and *Icmt*^*−/−*^ (N1, N2, and N3) isogenic cell lines. **(B)** Immunoblot analysis of MAPK activation, DNA damage, and apoptosis in the lysates of MDA-MB231 parental cells grown in the soft agar under the treatment of PD184352, a MEK inhibitor, at the indicated concentrations. **(C, D)** Soft agar colony formation on MDA-MB231 cells treated with different concentrations of PD184352. The scale bar length = 2 mm in the images. **(D)** Quantification of the colonies in (C) using OpenCFU and Prism5 software. “***”*P* < 0.0001. **(E)** Immunoblot analysis of AKT activation, DNA damage, and apoptosis in the lysates of MDA-MB231 parental cells grown in the soft agar under the treatment of Triciribine, an AKT inhibitor, at the indicated concentrations. **(F, G)** Soft agar colony formation on MDA-MB231 cells treated with different concentrations of Triciribine. The scale bar length = 2 mm in the images. **(G)** Quantification of the colonies in (F) using OpenCFU and Prism5 software. There are no statistical differences among different treatment conditions. **(H, I)** Comet assay for DNA damage in response to MEK inhibitor treatment. MDA-MB231 cells were treated with PD184352 at the indicated concentrations for 48 h under normal culture condition before seeding for comet assay. The TailMoment (D) were calculated using Casplab software based on the analysis of more than 100 cells for each PD184352 concentration—two representative images for each condition are shown in (E). See the Materials and Methods section for details. “***”*P* < 0.001. The study was repeated with similar results. The scale bar length = 100 μm in the images. For all immunoblots, the numbers below each marker are the densitometry quantification of band intensity.

The data detailed above suggests that ICMT function is important for the DNA damage repair, cancer cell proliferation, and survival under anchorage-independent growth conditions. PI3K and MAPK signaling are two major oncogenic pathways, which are also downstream of oncogenic RAS. Hence, in the investigation for the mechanism underlying this impact on cancer cells, we studied the role of MAPK and PI3K signaling–in DNA damage repair and in mediating the ICMT regulation of such phenotypes. In the immunoblot analysis of the Icmt^+/+^ and Icmt^−/−^ isogenic cells, we observed that all major components of the MAPK pathway, namely cRAF, MEK, and ERK, had reduced levels of phosphorylation in the *Icmt*^*−/−*^ cells compared with *Icmt*^*+/+*^ cells (Fig 4A) consistent with an earlier observation of the impact of ICMT inhibition on pERK (68), whereas there were less consistent changes in the pAKT levels. However, the pAKT level assessment is limited by its very low expression level for antibody detection.

To further determine the signaling pathway, either MAPK or PI3K/AKT, which accounts for the differences in the abilities of *Icmt*^*+/+*^ and *Icmt*^*−/−*^ cells to carry out DNA damage repair, we treated MDA-MB231 parental cells with escalating concentrations of either the MEK inhibitor PD184352 or the AKT inhibitor Triciribine. We postulated that, p-γH2AX would accumulate in a dose-dependent manner in response to a pathway-specific inhibitor if the activation of the pathway is required for the DNA damage repair. Indeed, we found that PD184352 induced dose-dependent reduction of pERK correlated with the increase of p-γH2AX and cleaved caspase 7—markers for persistent DNA damage and the activation of apoptosis (Fig 4B). Phenotypically, PD184352 treatment resulted in dose-dependent reduction of soft agar colony formation (Fig 4C and D). In contrast, Triciribine inhibition of pAKT, the major PI3K downstream effector, was associated with neither changes of p-γH2AX or cleaved caspase 7 markers (Fig 4E), nor that of soft agar colony formation ability (Fig 4F and G). The differences between the cell responses to MEK inhibitor and ATK inhibitor treatment support the notion that the MAPK, not the PI3K/AKT signaling, plays a functionally important role in DNA damage repair in the breast cancer cells. Finally, the effect of MEK inhibitor on DNA damage was visualized by comet assay in MDA-MB231 cells. Consistent with the changes in p-γH2AX, the TaillMoment (Fig 4H) calculated from the fluorescent single cell DNA fragmentation imaging (Fig 4I) increased in a PD184352 dose-dependent manner, confirming the importance of ERK activity in DNA damage repair and survival of MDA-MB231 cells.

**Figure 5.**
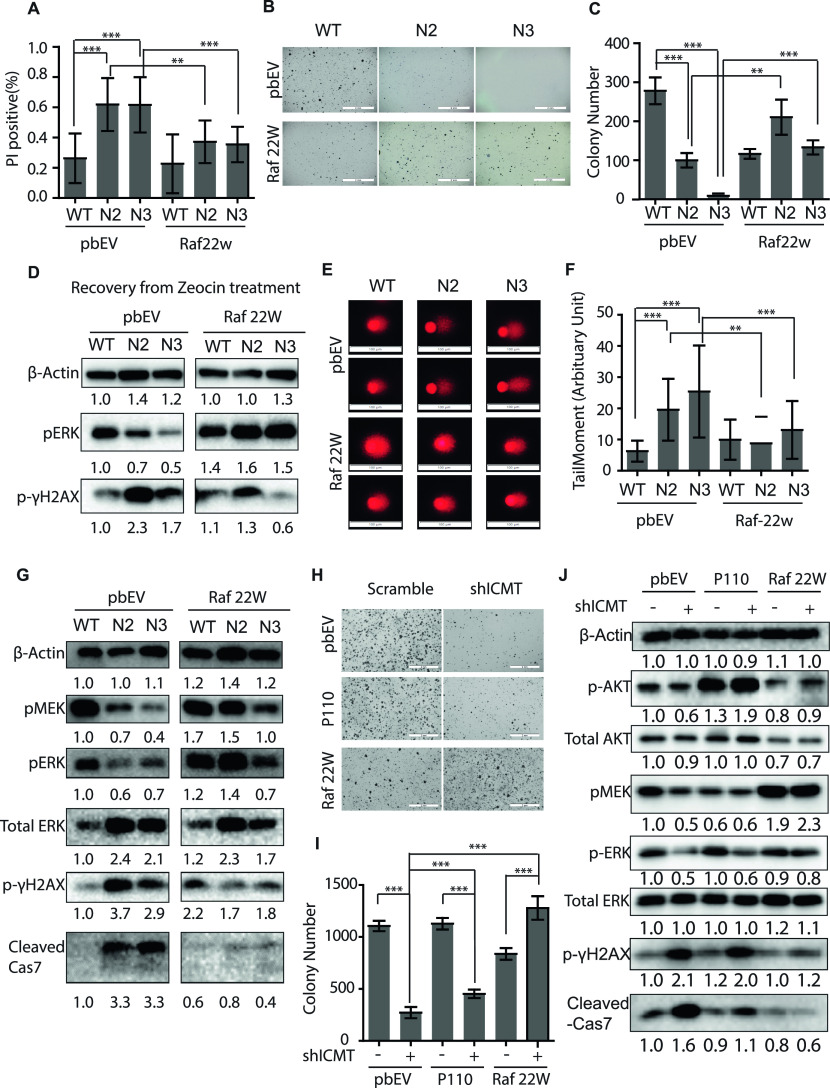
Expression of constitutively active cRAF reduces DNA damage and apoptosis in the *Icmt*^*−/−*^ cells, and restores their ability to grow in soft agar. **(A)** Propidium iodide staining and quantification for apoptotic cells among the *Icmt*^*+/+*^ (WT) and *Icmt*^*−/−*^ isogenic cells seeded in soft agar with and without the expression of RAF-22W, an activated form of cRAF. **(B, C)** Soft agar colony formation of the isogenic cells with and without the exogenous expression of RAF-22W. **(B)** Light microscopic images of the colonies, which were quantified in (C) using OpenCFU and Prism5 software. “**”*P* < 0.001; “***”*P* < 0.0001. The scale bar length = 2 mm in the images. **(D)** Immunoblot analysis of pERK and p-γH2AX in lysate of the isogenic cells with and without the exogenous expression of RAF-22W; the cells were pretreated with zeocin for 48 h followed by recovery in drug-free medium for 24 h. **(E)** Comet DNA damage assay of the isogenic cell lines with and without exogenous expression of RAF-22W. Cells were grown in suspension culture for 48 h before subjected the standard comet assay as described in the Materials and Methods section. Two representative images of cells in each group are shown; the scale bar length = 100 μm in the images. **(F)** Data analysis and quantification for TailMoments of the study shown in (E); 100 cells were analyzed for each group of cells. “**”*P* < 0.01; “***”*P* < 0.001. The experiment was performed three times with similar results. **(G)** Immunoblot analysis of MAPK signaling components, DNA damage and apoptosis markers in the isogenic cells grown in soft agar, with and without the exogenous expression of RAF-22W. Protein extraction from cells grown in soft agar is describe in detail in the Materials and Methods section. **(H, I)** Soft agar colony formation of MDA-MB231 cells stably expressing control shRNA or ICMT-targeting shRNA, with and without the exogenous expression of p110-CAAX or RAF-22W. **(H)** Light microscopic images of the colonies, which were quantified in (I) using OpenCFU and Prism5 software. “***”*P* < 0.0001. The scale bar length = 2 mm in the images. **(H, I, J)** Immunoblot analysis of markers for MAPK and AKT signaling, DNA damage and apoptosis in cells described in (H, I) grown in soft agar. Protein extraction from cells grown in soft agar is describe in detail in the Materials and Methods section. For all immunoblots, the numbers below each marker are the densitometry quantification of band intensity relative to the control.

The pathway-specific inhibitor treatment data support the conclusion that reduction of ERK activity compromises the ability of breast cancer cells to repair DNA damage, which is the likely mechanism for the accumulation of the damage and cell cycle arrest in *Icmt*^*−/−*^ cells. To further distinguish the roles of MAPK and PI3K signaling in DNA damage repair, we performed rescue experiments by increasing pathway-specific signaling via the expression of either constitutively active cRAF (RAF22W) or p110-CAAX in *Icmt*^*−/−*^ cells, and evaluated the impact on DNA damage, cell cycle progression, apoptosis, and colony formation ability. First, we found that expression of cRaf-22W significantly reduced apoptosis in *Icmt*^*−/−*^ cells cultured in soft agar (Fig 5A), and restored their colony formation ability (Fig 5B and C). To investigate whether expression of cRAF-22W enhanced DNA damage repair, we subjected the *Icmt*^*+/+*^ and *Icmt*^*−/−*^ cells, with and without exogenous expression of cRAF-22W, to treatment with low dose zeocin for 48 h followed by 24 h of release and determined the efficiency of DNA repair. In this setting, we observed that successful restoration of pERK in *Icmt*^*−/−*^ cells by expressing cRAF-22W is accompanied by the reduction of zeocin-induced *p*-γH2AX (Fig 5D). We also performed the comet assay to study the ability of cRAF-22W to rescue DNA damage repair in *Icmt*^*−/− *^cells cultured in suspension. Consistent with the other rescue results, the comet assay showed that expression of cRAF-22W reduced the TailMoment of *Icmt*^*−/−*^ cells to similar levels to those of the *Icmt*^*+/+*^ cells grown in suspension (Fig 5E and F), which is consistent with the notion that MAPK signaling is essential for DNA damage repair capability. Immunoblot analysis on the same cells used for comet assay demonstrated that cRaf-22W expression and accompanying increased levels of activated MEK and ERK led to significant reduction of *p*-γH2AX and cleaved caspase 7 (Fig 5G).

We next compared the effect of RAF22W and p110-CAAX over-expressing in restoring the soft agar colony formation ability of stable ICMT knockdown cells; we found that only the expression of RAF22W but not p110-CAAX restored the colony forming ability in ICMT knockdown cells (Fig 5H and I). Consistently, the immunoblot evaluation demonstrated that whereas both p110 and RAF22W sufficiently activated their downstream effectors—pAKT and pMEK, respectively, only RAF22W reduced the p-γH_2_AX and cleaved caspase 7 (Fig 5J). Worth noting, the ICMT knockdown cells instead of knockout cells were used here for the comparison of RAF22W and p110 overexpression effect, as we have observed significant toxicity of expressing p110 in the Icmt^−/−^ cells that was limiting their colony formation ability.

In summary, the studies of manipulating MAPK signaling in either direction using control and ICMT suppressing MDA-MB231 cells provide convincing evidence to support the importance of ERK activation in the repair of DNA damage, caused by either irradiation mimic agent zeocin or by anchorage-independent growth conditions. More importantly, these data establish that loss of *Icmt* compromise the cell machinery of DNA damage repair by suppressing the activity level of the MAPK signaling pathway.

### ICMT regulates the expression of key DNA damage repair pathway genes mediated through MAPK pathway

**Figure 6.**
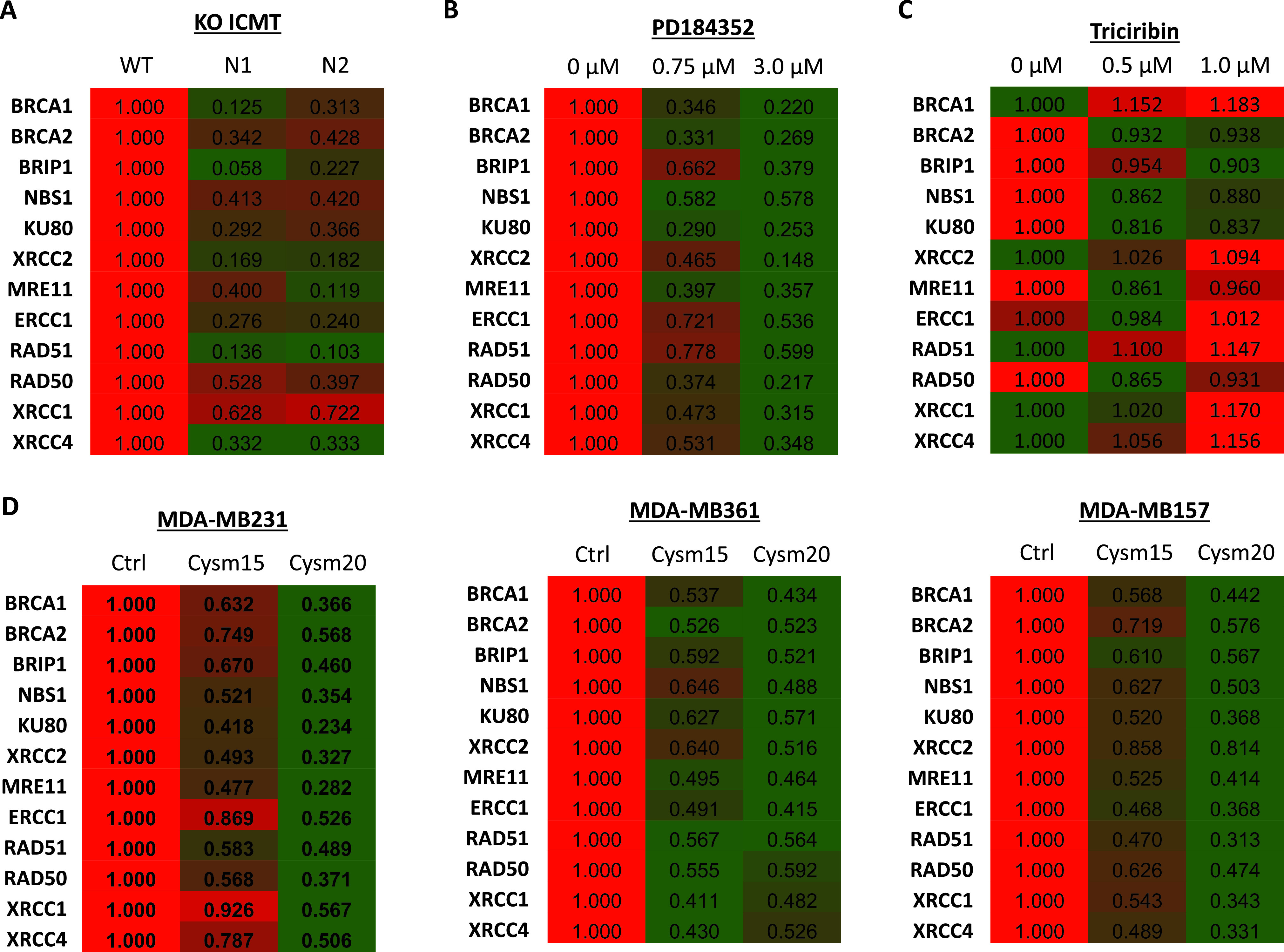
ICMT regulates the expression of multiple DNA damage repair genes through the regulation of MAPK signaling. **(A)** qPCR analysis of a panel of DNA damage repair genes in Icmt^+/+^ (WT) and Icmt^−/−^ (N2 and N3 mixed clones) MDA-MB231 cells. **(B, C)** Q-PCR analysis of the same panel of genes in MDA-MB231 cells treated with either vehicle control or two different concentrations of either PD184352 MEK inhibitor or Triciribine AKT inhibitor. **(D)** Q-PCR analysis of the same panel of genes in three different breast cancer cell lines after subjecting to either vehicle control or the indicated concentrations of cysmethynil ICMT inhibitor treatment.

**Figure S2.**
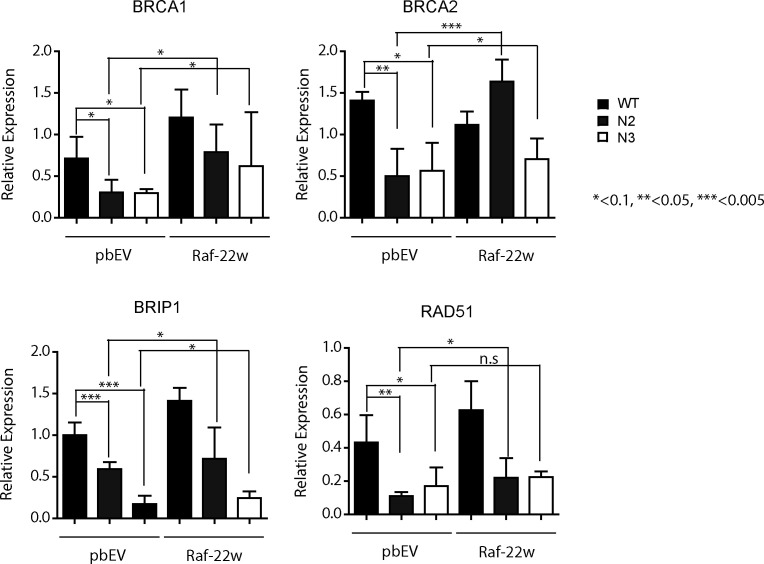
RAF22W expression rescues the expression of key DNA damage repair genes that are reduced as result of loss of ICMT. Q-PCR analysis of DNA damage repair genes BRCA1, BRCA2, BRIP1 and RAD51 in *Icmt*^*+/+*^ (WT) and *Icmt*^*−/−*^ (N2 and N3 mixed clones) MDA-MB231 cells, with or without the expression of constitutively active RAF–RAF22W. Black, grey and white bars represent the expression level in WT, N2, and N3 mixed clones, respectively. “*”, “**”, and “***” represent *P* < 0.1, *P* < 0.05 and *P* < 0.005, respectively.

We next evaluated the potential impact of ICMT on the DNA damage repair machinery. To this end, we assessed the transcription of several key proteins that are involved in various processes in DNA damage repair, including nucleotide excision, HR, non-homologues end joining, and single-strand break repair (19, 20, 69, 70, 71, 72, 73, 74, 75, 76, 77, 78, 79, 80, 81, 82, 83, 84). Expression analysis revealed that *Icmt*^*−/−*^ cells express significantly less of several key DNA repair genes compared with *Icmt*^*+/+*^ cells (Fig 6A). Consistent with the notion that MAPK signaling is the critical downstream mediator for this ICMT function, MEK inhibitor treatment similarly reduced the expression of the same genes in a dose-dependent manner (Fig 6B). In contrast, AKT inhibitor treatment did not result in similar gene expression changes, providing further evidence that AKT does not play a similar role in DNA damage repair in MDA-MB231 cells (Fig 6C). Further validating the role of ICMT in the regulation of the expression of these genes, we subjected multiple breast cancer cell lines to ICMT inhibitor treatment. Importantly, pharmacological inhibition of ICMT in multiple breast cancer cells resulted in a dose-dependent reduction of expression of the same panel of genes (Fig 6D). Finally, we assessed the impact of introduction of RAF22W on the expression of these DNA damage repair genes, which demonstrated consistently that activation of MAPK signaling via this route significantly increased the expression of several of the DNA damage repair genes (Fig S2), consistent with the results of MEK inhibitor treatment.

### Suppression of ICMT sensitizes breast cancer cells to PARP inhibitor–induced DNA damage and reduces their ability to form xenograft tumors

DNA damage occurs frequently in both benign and malignant cells. DNA damage triggers cell cycle check points and, ultimately, the programmed cell death pathway. PARP1 is involved in many aspects of DNA damage repair response, and has been particularly linked to the process of repair of single-strand DNA breaks (85); when PARP1 is inhibited, DNA damage accumulates (86). BRCA family of proteins are well-known for their roles in the homologous recombination repair pathway (87). BRCA loss-of-function mutations are predisposition factors for the development of breast and ovarian cancers (88). Indeed, cancers that carry BRCA mutations are more vulnerable, compared to the BRCA wild-type cancers, to PARP inhibitors because of their reduced ability to repair DNA damage.

**Figure 7.**
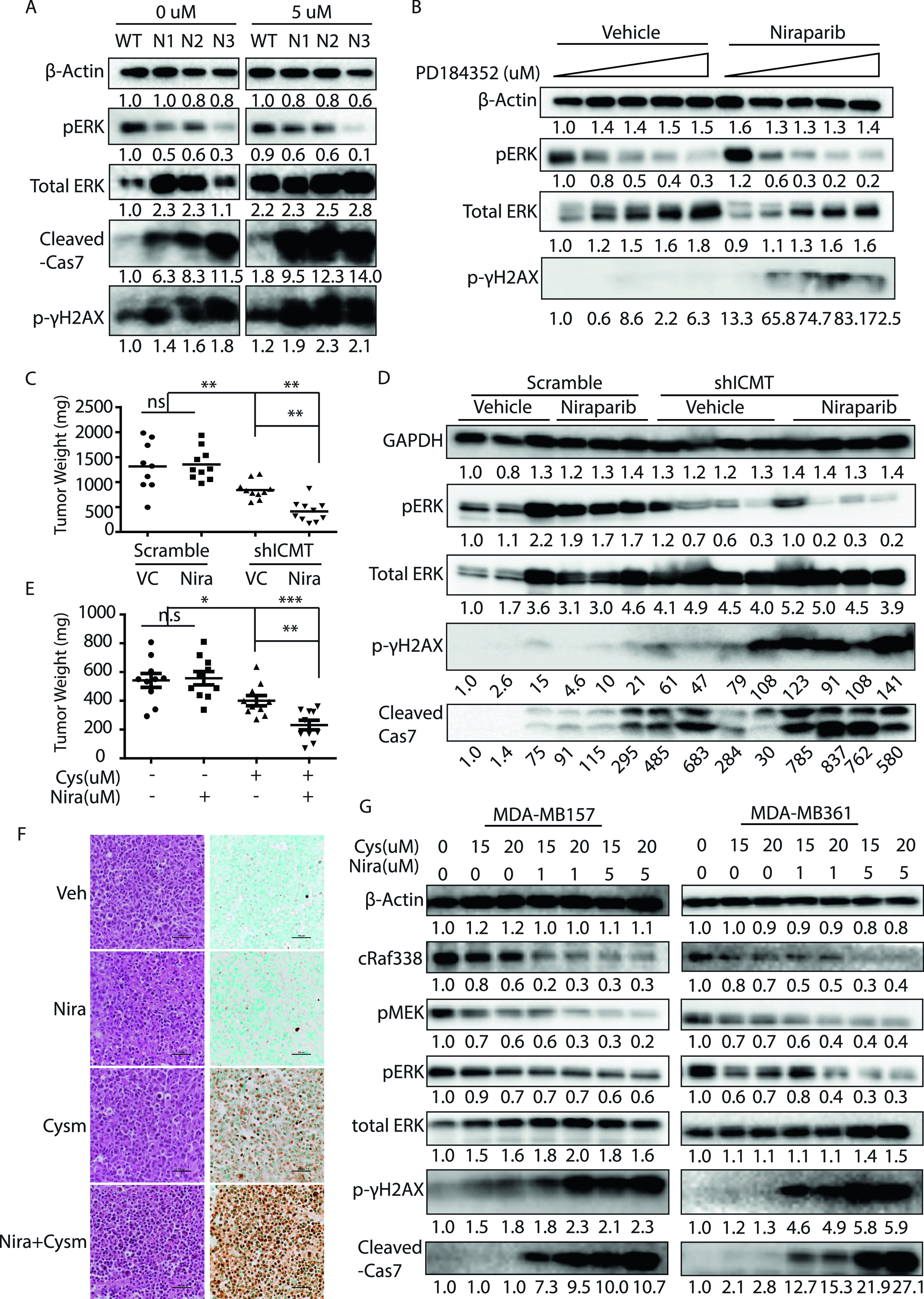
Concurrent suppression of ICMT and PARP1 is synergistic in eliciting DNA damage, inducing apoptosis and inhibiting tumor growth for MDA-MB231 breast cancer cells. **(A)** Immunoblot analysis of pERK markers, p-γH2AX, and cleaved caspase 7 in the lysates of *Icmt*^*+/+*^ (WT) and *Icmt*^*−/−*^ (N1, N2 and N3) isogenic cells treated with either vehicle control or 5 μM of niraparib 48 h under the standard culture condition. **(B)** Immunoblot analysis on the lysates of parental MDA-MB231 cells treated with either MEK inhibitor alone, or combination of the MEK inhibitor with 5 μM niraparib. Cells were pre-treated with MEK inhibitor PD184352 at the indicated concentration for 24 h, followed by the co-treatment with 5 μM niraparib for another 48 h, before lysate preparation for SDS–PAGE and immunoblot of the indicated proteins. **(C)** Xenograft tumor growth of implanted MDA-MB231 cells with and without ICMT knockdown, under either vehicle or niraparib treatment. Fifteen million cells expressing either control shRNA or ICMT-targeting shRNA were subcutaneously implanted in the flanks of SCID mice to form tumors. The tumor sizes of different groups at the time of treatment initiation are summarized in Fig S4A. Mice were treated with either vehicle control or 80 mg/kg niraparib and tumor growth monitored until termination of experiment. The tumor volumes of the indicated cell groups at the end of the study are graphed here. “**”*P* < 0.01. The study is detailed in the Materials and Methods section. **(C, D)** Immunoblot analysis for ERK activation, p-γH2AX, and cleaved caspase 7 on the lysates of the isolated tumors from (C). **(E)** Xenograft tumor growth of implanted MDA-MB231 cells treated with either vehicle control, 80 mg/kg niraparib alone, 100 mg/kg cysmethynil alone, or combination of niraparib and cysmethynil. Fifteen million MDA-MB231 parental cells were subcutaneously implanted in the flanks of SCID mice to form tumors. The tumor sizes of different groups at the time of treatment initiation are summarized in Fig S5A; tumor growth was monitored until termination of experiment (Fig S5B). The tumor volumes of the indicated cell groups at the end of the experiment are graphed here. “*”*P* < 0.05; “**”*P* < 0.005; “***”*P* < 0.0005. **(E, F)** TUNEL Assay to quantify apoptosis in the tumors isolated from (E). The left column: H&E analysis images; the right column: TUNEL assay images; the four rows from top to bottom are vehicle, niraparib, cysmethynil, and combination treatment groups, respectively. Scale bar: 100 μm. **(G)** Immunoblot analysis on breast cancer cell lines MDA-MB157 and MDA-MB361 that are subjected to the treatment of either cysmethynil alone or combination of cysmethynil and niraparib. The cells were treated for 48 h before lysate preparation for SDS–PAGE and immunoblotting for the indicated proteins. For immunoblots, the numbers below each marker are the densitometry quantification of band intensity. For all immunoblots, the numbers below each marker are the densitometry quantification of band intensity relative to the control.

**Figure S3.**
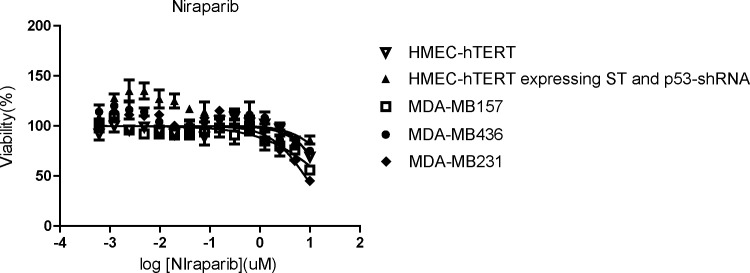
Dose response viability analysis of mammary epithelial and multiple breast cancer cells after 3-d treatment of niraparib. The cells were seeded at 3,000 cell/96 well density and left for attachment overnight, before treated by indicated concentration of niraparib for 3 d and MTS substrate solution was added for colormetric analysis. The readings were analyzed by Prism5. The Means and SD were calculated from triplicate wells; the study was repeated with similar results.

MDA-MB231 is among the breast cancer cell lines that are considered resistant to PARP inhibitor treatment (89). To further explore the notion that ICMT function is essential in supporting ERK-dependent DNA damage repair and the potential application in cancer treatment, we evaluated the effect of concurrent suppression of PARP1 and ICMT. We postulated that, based on current evidence, suppression of ICMT would render the usually resistant MDA-MB231 cells into a “BRCA-like” state, hence vulnerable to the treatment with PARP inhibitor. To this end, we treated *Icmt*^*+/+*^ and *Icmt*^*−/−*^ cells with either vehicle control or the PARP1 inhibitor niraparib for 48 h, followed by immunoblot assessment of the levels of p-γH2AX and cleaved caspase 7 for DNA damage and apoptosis. We found that these markers only slightly elevated in the *Icmt*^*+/+*^ MDA-MB231 cells in response to niraparib treatment (Fig 7A). Consistent with the sluggish marker response, niraparib treatment had little effect on cell proliferation or survival, as assessed by cell viability assay, on multiple breast cancer cell lines (Fig S3). In the *Icmt*^*−/−*^ cells, however, the baseline levels of p-γH2AX and cleaved caspase 7, which were higher than *Icmt*^*+/+*^ cells, were further elevated in response to niraparib treatment (Fig 7A). Next, we assessed the role of ERK activity in mediating the combination effect of ICMT and PARP suppression by co-treatment of the *Icmt*^*+/+*^ cells with MEK and PARP inhibitors. Here, we found that inhibition of MEK or PARP1 alone resulted in only slight elevation of p-γH2AX, whereas the combination of low dose of MEK inhibitor with 5 μM of niraparib led to massive elevation of p-γH2AX (Fig 7B). Noteworthy, the reduction of pERK under MEK inhibitor treatment is at similar level with and without niraparib, consistent with the understanding that the two inhibitors work by independent mechanisms. This combination study not only supports the role of ERK activity in ICMT regulation of DNA damage repair activity, but also demonstrates clearly that combined inhibition of MAPK pathway and PARP1 function can be potentially useful in the treatment of this group of cancers.

**Figure S4.**
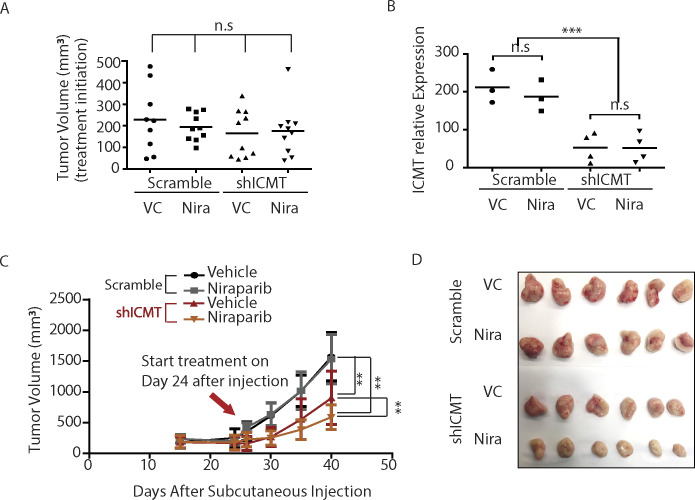
Tumor growth inhibition study of combining ICMT knockdown and PARP inhibitor. **(A)** Tumor volumes of different cell groups at the time of drug treatment initiation. VC, vehicle control; Nira, niraparib. “n.s.”, not significant. **(A, B)** ICMT transcript levels determined by q-PCR on the isolated tumor tissues from the same in vivo groups as (A). **(A, B, C)** Growth curves of tumors of the same groups as in (A) and (B). **(A, B, C, D)** Images of the isolated tumors from the same groups as in (A), (B) and (C).

**Figure S5.**
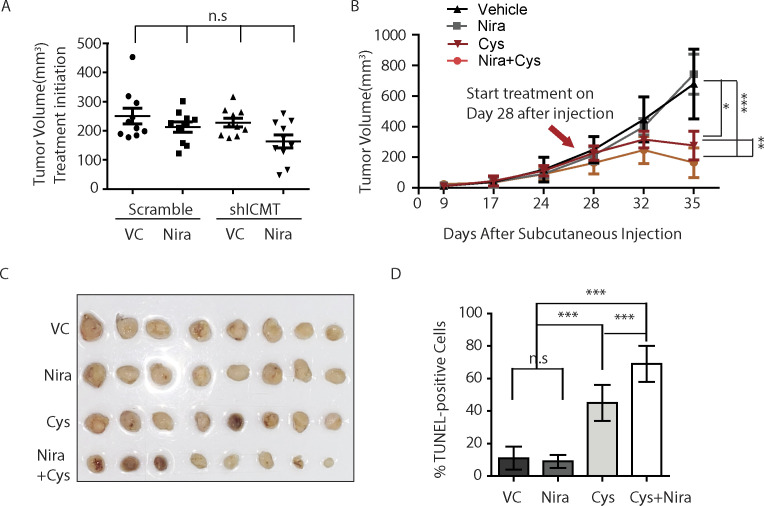
Tumor growth inhibition study of combining ICMT inhibitor and PARP inhibitor treatment. **(A)** Tumor volumes of different cell groups at the time of drug treatment initiation. VC, vehicle control; Nira, niraparib; Cysm, cysmethynil; “n.s.”, not significant. **(A, B)** Growth curves of tumors of the same groups as in (A). **(B, C)** Images of the isolated tumors from the same groups as in (B). **(C, D)** Quantification of TUNEL Assay positive cells using the tumor tissue preparations from (C), and graphed by Prism software. “n.s.” not significant; “***”*P* < 0.0005.

We further investigated the combination effect of PARP inhibition and ICMT suppression in in vivo models of tumor growth. The extent of suppression for individual target, that is, ICMT and PARP, was kept at moderate level, so as to allow potential synergy to be readily observable. To achieve moderate inhibition of ICMT, ICMT stable knockdown cells instead of knockout cells were used in the combination study. To this end, MDA-MB231 cells that express either control shRNA or ICMT-targeting shRNA were implanted in the flanks of SCID mice; the mice in each group were divided into vehicle or niraparib treatment sub-groups. Niraparib treatment at 80 mg/kg/day was initiated when the tumors had achieved the average sizes of 150–250 mm^3^ for all groups (Fig S4A). Tumor growth was monitored throughout the niraparib treatment course until the end of experiment, which was determined as when the fastest growing group of tumors reached the average size of 1.5 cm^3^ as required by IACUC protocol. RT-PCR validation of ICMT expression levels in the isolated tumors demonstrated that the ICMT level was maintained 60–70% of the control samples, which was desirable for the combination study; and there is no significant difference of ICMT expression between the vehicle and the niraparib treatment groups (Fig S4B). Analysis of tumor size showed that, despite being statistically indistinguishable among the four groups before the initiation of niraparib treatment (Fig S4A), the sizes of the tumors derived from ICMT knockdown cells were significantly smaller than those from control shRNA expressing cells at the end point (Figs 7C and S4C). More importantly, niraparib treatment further reduced the sizes of tumors derived from ICMT knockdown cells, whereas the same treatment had no effect on the tumors derived from cells expressing control shRNA (Figs 7C and S4C and D). The resistance to niraparib is expected because MDA-MB231 breast cancer cells are considered BRCA wild type, or in a broad and precise term, to have no deficiency in DNA damage repair machinery. As anticipated, immunoblot analysis on the tumor samples showed that niraparib treatment induced slight increase of p-γH2AX and cleaved caspase 7 in control tumors, whereas the same treatment led to massive elevation of these markers in the tumors derived from ICMT knockdown cells (Fig 7D). It is interesting to note that ICMT knockdown alone induced significant level of p-γH2AX and cleaved caspase 7, and reduced pERK consistent with the in vitro observations (Fig 7D). So far, the in vivo ICMT inhibition has been achieved by shRNA knockdown. However, it is important to evaluate whether pharmacological inhibition of ICMT has the same effect for potential future therapeutic considerations. To this end, we performed the similarly designed in vivo tumor growth inhibition study using ICMT small molecule inhibitor and niraparib. We observed consistent tumor growth inhibition patterns for vehicle control, either inhibitor alone and combination treatment, as that from the ICMT knockdown and niraparib combination study (Figs 7E and S5A–C). Furthermore, we performed TUNEL assay using the isolated tumors to study the effect of drug treatment on apoptosis. Consistent with the tumor growth pattern of the four groups, TUNEL assay showed that niraparib induced no changes in apoptosis, whereas cysmethynil and combination treatment induced significantly higher and massive levels of apoptosis, respectively (Figs 7F and S5D). Together, the in vitro and in vivo evidence lead us to conclude that ICMT suppression in breast cancer cells creates a “BRCA-like” state, hence sensitizing them to PARP1 inhibitor–induced growth inhibition and apoptosis.

Finally, we expanded the synthetic lethality study of ICMT and PARP1 inhibition to other breast cancer cells. To this end, we subjected additional breast cancer cell lines to cysmethynil or niraparib or combination treatment. We observed that cysmethynil treatment, similar to genetic knockdown of ICMT, reduced ERK activation; and the combination of cysmethynil and niraparib resulted in robust DNA damage and apoptosis (Fig 7G).

## Discussion

**Figure S6.**
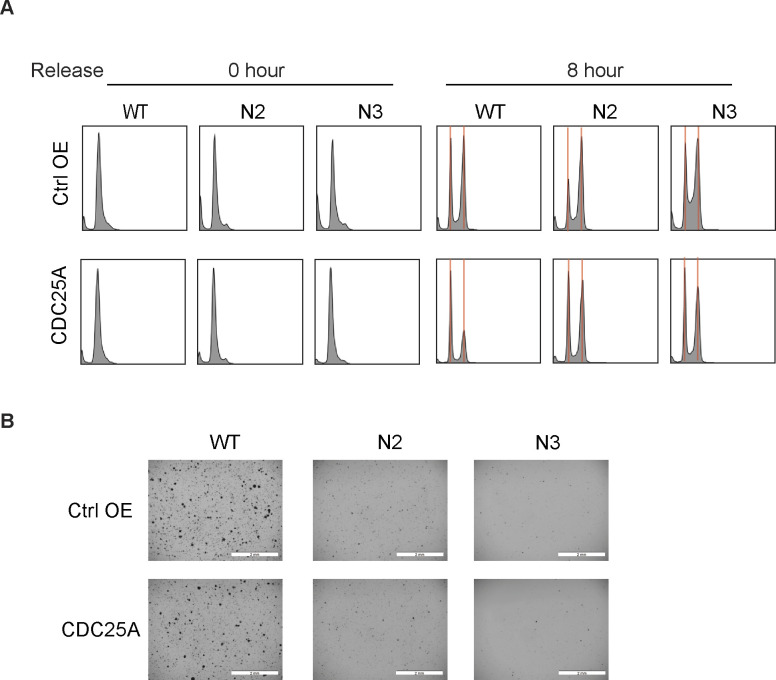
Overexpression of CDC25A corrects the G2/M arrest in Icmt^−/−^ MDA-MB231 cells but does not restore the cells’ ability to growth in soft agar. **(A)** Cell cycle distribution of the *Icmt*^*+/+*^ and *Icmt*^*−/−*^ isogenic MDA-MB231 cells, with or without over-expressing CDC25A, following double thymidine synchronization (0 h) and 8 h post-release. The red vertical lines mark the G1 and G2/M peaks. The cells were prepared by standard propidium iodide staining method for cell cycle analysis, as described in the Materials and Methods section. **(B)** Soft agar colony formation of the isogenic cells with and without exogenous expression of CDC25A. The study has been repeated 2 times with similar results. Scale bar length = 2 mm.

A role for ICMT in regulating DNA damage repair has not been previously recognized, despite multiple reports of cell cycle arrest associated with ICMT inhibition (48, 90). In this study, we observed significant increase of G2/M population in the *Icmt*^*−/−*^ MDA-MB231 cells compared with the parental cells, leading us to examine the process of DNA damage and repair. We first evaluated whether ICMT directly affects G2/M check point proteins to cause the arrest or DNA damage accumulation leads to secondary G2/M arrest. CDC25 proteins are critical components of the G2/M check point and had been introduced in MDA-MB231 cancer cells to reverse G2/M arrest and rescue cell death in various studies (91, 92). To investigate whether loss of ICMT function directly impacts the G2/M check point, we overexpressed the functionally important CDC25A in the *Icmt*^*−/−*^ cells to evaluate the rescue effects. We observed that although CDC25A overexpression significantly reduced the population of cells in G2/M (Fig S6A), the ability of the *Icmt null* cells to form colonies in the soft agar was not at all affected (Fig S6B), suggesting that the G2/M arrest is not the root cause for cell death and loss of anchorage-independent growth for the *Icmt null* cells. In other word, simply forcing *Icmt null* cells to pass the check point would not rectify the consequence from the loss of ICMT function.

**Figure 8.**
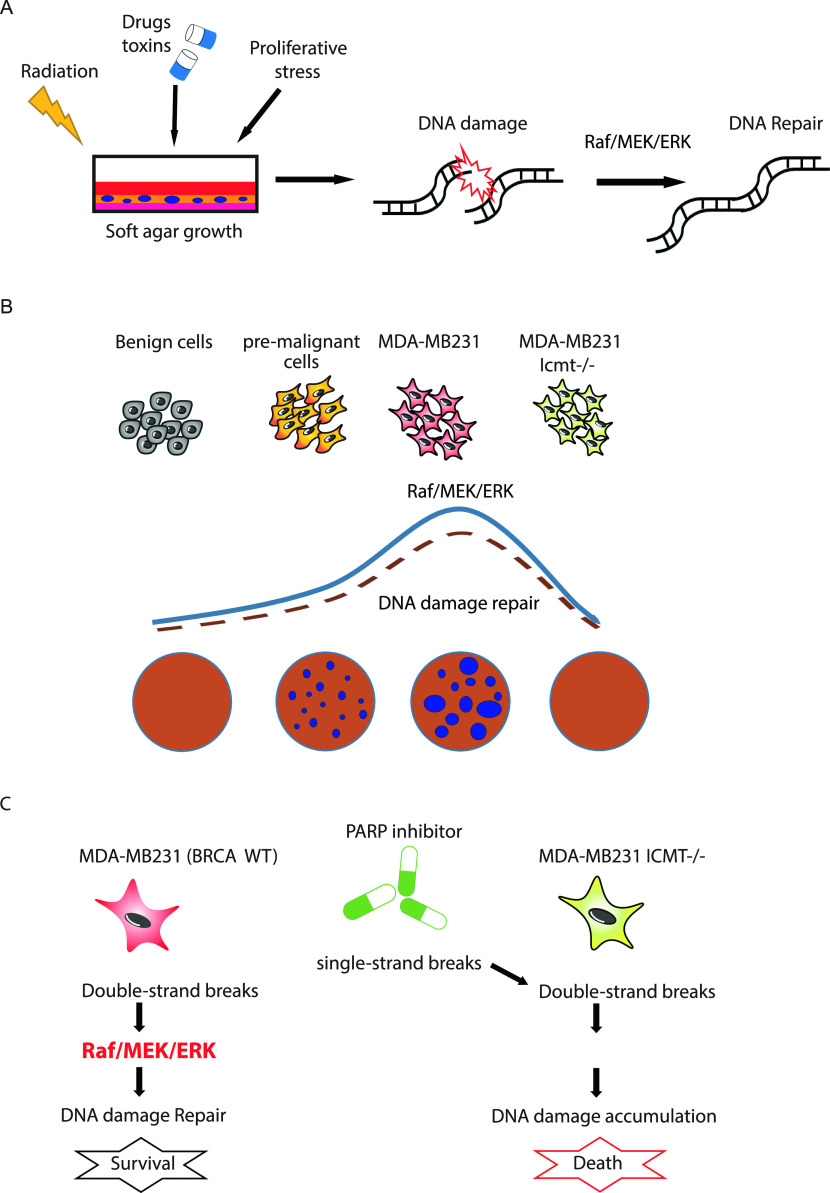
Schematic summary of key findings from the study. **(A)** Exposure to drug/toxin, irradiation and suspension growth condition all cause DNA damage; the repair of the DNA damage requires active MAPK signaling. **(B)** Malignant transformation in many cancers, such as in breast epithelial cells, increases the activation of MAPK signaling, which enhances the cells’ ability to repair DNA damages and survive in suspension culture. Loss of ICMT function inhibits the MAPK activation, which impairs the DNA damage repair and increases apoptosis, leading to the abolishment of colony and tumor formation. **(C)** Loss of ICMT function in MDA-MB231 cells impairs DNA damage repair and renders them susceptible to PARP inhibitor treatment.

DNA damage is one of the major triggers for cell cycle arrest (93). It is understood that halting of proliferation in the presence of DNA damage is necessary for multicellular organisms to maintain genetic integrity. DNA damages can be induced by many physical, chemical and biological factors, most notably radiation, drugs/toxins and replicative stress (4, 5, 53, 94, 95) (Fig 8A). Many cancer cells have sufficient DNA damage repair function that preserves their survival and proliferative ability; however, acute increase in DNA damage or reduction of the capacity for repair would lead to fatal accumulation of DNA breaks. In our study, we found that loss of ICMT function results in the accumulation of DNA damages, leading to G2/M arrest and apoptosis.

The accumulation of DNA damage associated with loss of ICMT could either be the result of elevated damage-inducing stresses or decreased repair capability. To distinguish the two possibilities, we treated both *Icmt*^*+/+*^ and *Icmt*^*−/−*^ cells with irradiation mimic drug zeocin to induce DNA damage and then observed the rate of damage repair during drug-free recovery period. Using comet assay and DNA damage marker analysis, the post zeocin recovery study led us to conclude that ICMT function is important for the repair of DNA damage. Interestingly, we found that in addition to the usual stressors such as irradiation and toxins, growing under suspension condition is an independent inducer for DNA damage. In fact, the accumulation of p-γH_2_AX in the *Icmt*^*−/−*^ cells was most pronounced only when the cells were cultured in soft agar condition.

**Figure S7.**
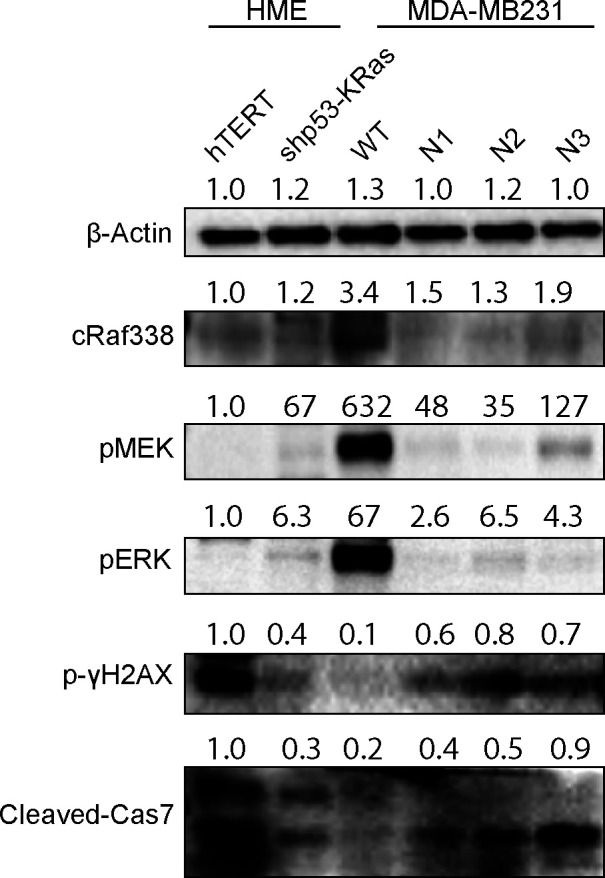
Malignant transformation is accompanied by increased MAPK signaling activity and lower level of DNA damage and apoptosis in breast epithelial cells, which are reversed by suppression of ICMT.

In the investigation for the mechanism of ICMT regulation of DNA damage repair, we looked at major signaling pathways in cancer, particularly the ones involving key ICMT substrates; the reduction of MAPK signaling is the most consistent when ICMT is suppressed in MDA-MB231 isogenic cells (Fig 4A), which is cogent with the fact that it is directly under the regulation of RAS oncogenes—well-known ICMT substrates (42, 45, 68). Up to now, the roles of MAPK in DNA damage and survival in cancer cells are not well-defined. Some studies have shown that activation of ERK, either from chemotherapy or from radiation, induced DNA damage (52, 53, 54, 57, 94). In these settings, suppressing ERK activation reduced cell death induced by DNA damage (96, 97). Yet, other studies demonstrated that ERK is important for HR DNA repair, which is compromised by loss of ERK activation (58, 59). In the current study, we observed that maintaining MAPK activation is critical for the DNA damage repair in breast epithelial cancers, assessed by multiple breast cancer cell lines; the much suppressed MAPK signaling from the loss of ICMT leads to marked accumulation of DNA damage and apoptosis. Further strengthening the relevance of MAPK signaling in ICMT-dependent DNA damage repair, expression of constitutively activate cRAF-22W in the *Icmt null* cells restored DNA damage repair capacity, reduced apoptosis and restored cells’ ability to form colonies in soft agar (Fig 5). Consistently, MEK inhibitor treatment of MDA-MB231 cells induces the accumulation of DNA damage, G2/M arrest and apoptosis in a dose-dependent manner in soft agar (Fig 4). Finally, in the telomerase immortalized human mammary epithelial (HME-hTERT) cells, we observed that the transformation state induced by p53 knocking-down and mutant RAS expression (HME-shp53-Ras) increases MAPK signaling and reduces pγH2AX and cleaved caspase 7 cleavage. In the established cancer cell line MDA-MB231, which has a highly activated MAPK pathway, loss of ICMT reduced the MAPK activation leading to DNA damage accumulation and apoptosis (Fig S7). Hence, we postulate that MAPK pathway promotes malignant transformation by maintaining DNA damage repair capability and safe guard cell survival (Fig 8B).

It is widely recognized that accumulation of a significant amount of damaged DNA is a trigger for the halting of cell cycle progression and induction of apoptosis (98). Thus, measures that increase DNA damage and/or decrease the cells’ capacity for DNA repair have potential therapeutic benefit. In this regard, PARP1 inhibitors compromise the repair of DNA breaks leading to the accumulation of double-strand DNA breaks in proliferating cells (17, 18). Although it is increasingly recognized that PARP1 inhibitors suppress DNA repair in multiple ways, they generally work better through the mechanism of synthetic lethality with DNA repair deficiencies, endogenous or induced (99, 100). The efficacy of PARP inhibitors is much diminished in cancers that have intact, or even enhanced ability for DNA repair and tolerability for genomic instability (12, 101). Therefore, for many cancers the synthetic lethality needs to be created by combining PARP1 inhibition with an agent that compromises DNA damage repair or increases DNA damage (102) (Fig 8C). The evidence presented in this study has identified a novel function of ICMT in DNA damage repair through its regulation of MAPK signaling. From this standpoint, it is a potential strategy to induce DNA damage and apoptosis by ICMT inhibition, either as a single agent or in combination with DNA damage–inducing agents such as PARP inhibitors or irradiation to achieve broader efficacy against resistant cancer cells (Fig 8C).

## Materials and Methods

### Cell culture

MDA-MB231, 436, 157, 361, and 453; HCC38, HME-1; and HEK293T cells, obtained from American Type Culture Collection, were cultured in high glucose DMEM or RPMI1640 (HCC38) containing 10% fetal bovine serum and 1% penicillin/streptomycin. Human mammary epithelial (HME-1) cells that express human telomerase (HME-1-hTERT), both human telomerase and SV40 small T antigen (HME-1-ST), and HME-1-ST with additional shRNA targeting p53 and expressing mutant KRAS (HME-1-shp53-kRAS) were cultured as previously described (Lau et al, 2017) (44). For soft gar colony formation assay, low-gelling-temperature agarose, noble agar, DMEM—high glucose powder, PI and methylthiazolyldiphenyl-tetrazolium bromide (MTT) from Sigma-Aldrich were the reagents used. The bottom agar layer contains DMEM, 10% FBS, and 0.5% noble agar. The cells are mixed with 0.25% noble agar DMEM with 10% FBS. For the HME cells, the media on top was DMEM plus 10% FBS, 0.5 μg/ml hydrocortisone and 5 μg/ml insulin, whereas for MDA-MB231 cells it was DMEM and 10% FBS. Cells were maintained in such condition for 2–3 wk and then developed by incubating in 0.2 mg/ml methylthiazolyldiphenyl-tetrazolium bromide (MTT) solution for 3–5 h at 37°C. The colonies were visualized and imaged by Olympus SZX16 Research Stereo Microscope with a 2.5× objective. More than five randomly chosen views were analyzed for each colony formation conditions using OpenCFU software.

### Reagents and antibodies

The MEK inhibitor PD184352, Bleomycin, Thymidine, Puromycin Hydrocortisone, and PI were purchased from Sigma-Aldrich; Hygromycin was from Invitrogen; Insulin was from I-DNA Biotech; and Triciribine (S1117) and niraparib (HY-10619) were purchased from Selleckchem and MCE (MedChemExpress), respectively; cysmethynil was synthesized by Duke University Small Molecule Synthesis Facility. All antibodies, including those for β-actin, pMEK (#9121), MEK, pERK (#4377), ERK, cleaved Cas7 (#8438), p-γH2AX (#9718), pCDC2 (#4539), total-CDC2 (#28439), cyclin B1 (#4135), GAPDH (#5174), phospo-DNAPKs (2056), cyclin D1 (#2922), pAKT (#9271S), and total AKT (#9272S) were purchased from Cell Signaling Technology. TUNEL Assay Kit (ab206386) for tissue apoptosis study was from Abcam and used per manufacture’s protocol.

### Cloning of shRNA and cDNA into lentiviral and retroviral vectors and selection of stable cell lines

shRNAs targeting ICMT were cloned into lentiviral vector pLL3.7, whereas the cDNA encoding constitutively active c-RAF (RAF-22W) and p110-CAAX were cloned into pBabe-Puromycin vector. Lentivirus preparation and stable cell line selection are described in our previous publications (44, 47, 103). PHR’CMV Citrine–geminin and PHR’CMV mCherry-cdt1 were purchased from Addgene; retrovirus preparation was as described previously (44).

### Immunoblot and quantitative-real time polymerase chain reaction analysis

The cells were harvested in RIPA lysis buffer (Thermo Fisher Scientific) containing protease and phosphatase inhibitors (Sigma-Aldrich). Cell lysates were separated by standard SDS–PAGE, transferred to PVDF-FL membranes, blotted with primary antibody solution in PBST overnight at 4°C, washed three times with PBST, and then incubated in respective secondary antibodies at 1:10,000 at room temperature for 1 h. Membranes were washed multiple times with PBST before visualization using the Thermo Fisher Scientific SuperSignal West Femto Substrate (Thermo Fisher Scientific) and Bio-Rad ChemidoC. The images were analyzed and presented using the ImageLab software.

For PCR analysis, cDNA was generated from RNA samples using iScript cDNA Synthesis Kit (Bio-Rad). qRT-PCR was performed using the Thunderbird SYBR qPCR Mix (Toyobo) and the CFX96 Real Time System (Bio-Rad), followed by gene expression data analysis using the comparative CT method.

Primers used for the qPCR are listed below (from 5′ to 3″):

BRCA1-F: CTGAAGACTGCTCAGGGCTATC, BRCA1-R: AGGGTAGCTGTTAGAAGGCTGG;
BRCA2-F: GGCTTCAAAAAGCACTCCAGATG, BRCA2-R: GGATTCTGTATCTCTTGACGTTCC;
BRIP1-F: TCTGGAGTTGGTGAAGACAGTCA, BRIP1-R: CCACGACAAACTGCTACCAGGA;
NBS1-F: TCTGTCAGGACGGCAGGAAAGA, NBS1-R: CACCTCCAAAGACAACTGCGGA;
KU80/XRCC5-F: GCAGTGTCACCTCTGTTGGA, KU80/XRCC5-R: GCTCGGATGCAGTCTATGCT;
XRCC2-F: TCTGTTTGCTGATGAAGATTCACC, XRCC2-R: CATCGTGCTGTTAGGTGATAAAGC;
MRE11-F: GCCTTCCCGAAATGTCACTA, MRE11-R: TTCAAAATCAACCCCTTTCG;
ERCC1-F: CGGCGGAAACTCATCCGATA, ERCC1-R: CCATCAGGGCCTCCTCAAAG;
RAD51-F: TCTCTGGCAGTGATGTCCTGGA, RAD51-R: TAAAGGGCGGTGGCACTGTCTA;
RAD50-F: GCGGAGTTTTGGAATAGAGGAC, RAD50-R: GAGCAACCTTGGGATCGTGT;
XRCC1-F: TCTCCCGGGTGACTGAATGTC, XRCC1-R: CCCCAACTCCTTGGGTTCTT;
XRCC4-F: TGGACTGGGACAGTTTCTGA, XRCC4-R: TCAGTTCACCAACATATTTCCC.

### Thymidine cell synchronization assay

Cells grown to 30–40% confluency were treated with 2 mM thymidine in DMEM medium for 14 h in 37°C cell culture incubator. After PBS washing, the cells were then cultured in the standard DMEM medium for 9 h. The second thymidine block was started by incubating the cells in DMEM with 2 mM thymidine for 14 h, followed by the culturing in the normal DMEM medium for the release step. Cells were harvested by trypsinization after 0, 2, 4, and 8 h of release. Subsequently, the harvested cells were washed twice with PBS, fixed in 70% ethanol in 4°C overnight, treated with 200 μg/ml RNase A in PBS at 37°C for 30 min, stained with 50 μg/ml PI in PBS overnight, and then analyzed by MACSQUANT Flow Cytometry for cell cycle distribution. The data analysis was carried out using FlowJo-10 software.

### Analysis of DNA damage by alkaline lysis comet assay

Cells ready for the assessment, after appropriate treatment for each experiment, were embedded in 1% low-gelling-temperature agarose at 20,000/ml on the iBID chamber slides (#80426; iBIDI GmbH), which was submerged in the lysis buffer (1.2 M NaCl, 100 mM sodium EDTA, 0.1% sodium lauryl sarcosinate, and 0.26 M NaOH) overnight at 4°C in the dark. After the lysis step, the slides were washed three times with running solution which contained 0.03 M NaOH and 2 mM Na_2_EDTA in Milli Q water. The slides were then submerged in the electrophoresis chamber filled with running buffer and electrophoresed at 40 mA for 20 min, followed by washing in Milli Q water for three times, before staining with 2.5 μg/ml PI for 15 min. The cells in the gel were imaged using Olympus IX71S1F3 Fluorescent Microscope. Data were analyzed by Casplab; at least 200 cells were analyzed for each condition.

### Macromolecule extraction from cells embedded in soft agar

Protein and RNA extractions from cells embedded in soft agar were performed as recently detailed (104). Briefly, for protein analysis the cells were extracted with PBS and then snap frozen in liquid nitrogen and stored in −80°C for further processing. The thawed pellet was mixed with RIPA buffer containing protease and phosphatase inhibitors at 1 to 0.5 volume ratio; β-mercaptoethanol was added to 2% final concentration and the samples are heated at ≥95°C for 10 min, cooled on ice for 1 h, and centrifuged at 15,000*g* for 30 min at 4°C. The supernatant was mixed vigorously with 1 volume of 100% methanol and 0.25 volume of chloroform, and the precipitated protein was pelleted. Pellets were air-dried and resuspended in the appropriate amount of RIPA buffer + protease and phosphatase inhibitors for subsequent analysis.

For RNA extraction, the layer of agar containing the cells was homogenized in a 0.4 volume ratio of pre-warmed (65°C, in the dark) CTAC buffer, which contains 2% wt/vol cetyltrimethylammonium chloride (CTAC; Sigma-Aldrich), 2% wt/vol polyvinylpyrrolidone (PVP-40; Sigma-Aldrich), 2 M sodium chloride, 100 mM Tris–HCl at pH 8.0, 20 mM EDTA (Sigma-Aldrich); an equal volume of chloroform (Sigma-Aldrich) was then added and mixed vigorously. The mixture was centrifuged at 15,000*g* for 5 min at room temperature, and the top layer removed and mixed with an equal volume of isopropanol to precipitate nucleic acid. After centrifugation at 15,000*g* for 15 min at room temperature, the nucleic acid pellet was washed with 70% ethanol, pelleted at 15,000*g* for 5 min, and air-dried. The pellet was resuspended in 50 μl RNase-free water and incubated with 1 μl DNase I (Thermo Fisher Scientific) at 37°C for 30 min. RNA was collected using the FavorPrep Tissue Total RNA Mini Kit (Favorgen, state, country) following the modified protocol as recently reported (104). To obtain DNA, RNase-free water was used to resuspend air-dried nucleic acid pellet, which then was incubated with RNase A (QIAGEN) at 37°C for 30 min to degrade RNA. DNA was collected using the DNeasy Blood and Tissue Kit (QIAGEN) per manufacture’s protocol.

### Xenograft mouse model in vivo study and tumor tissue analysis

To generate xenograft tumors, 15 million MDA-MB231 breast cancer cells were subcutaneously implanted in the flanks of female SCID mice 8 wk of age, followed by tumor growth monitoring. Mice were orally dosed with either vehicle, 80 mg/kg niraparib daily, IP dosed 100 mg/kg cysmethynil every other day, or the combination of same doses of niraparib and cysmethynil for the treatment, as specified by each study, starting when the tumors reached the stable volume of 100–500 mm^3^. Tumor growth was followed until termination of experiment, which was when the fastest growing group of tumors, in this case the vehicle-treated control group, reached the mean size of 1,500 mm^3^. The study protocol was approved by the institutional Animal Care and Use Committee (IACUC). The tumors were isolated after mouse euthanization for imaging and sample preservation, which is by the standard fixation (HT501128; Sigma-Aldrich) and paraffin embedding method. Histology slide preparation and H&E staining were performed by Duke-NUS Histology Service. TUNEL assay to visualize apoptotic cells was per protocol of the manufacture of TUNEL Assay Kit (ab206386; Abcam).

### Statistical analysis

All the statistical analysis in this study was performed using GraphPad Prism software; data are presented as mean ± SD. To calculate the statistical significance, experimental groups were compared with the control group using Dunnett’s test one-way ANOVA to generate *P*-values. Statistical significance was defined as *P* < 0.05.

## Data Availability

All data that support the findings of this study are openly available or on request from the corresponding author.

## Supplementary Material

Reviewer comments
